# Stability Study of the Irradiated Poly(lactic acid)/Styrene Isoprene Styrene Reinforced with Silica Nanoparticles

**DOI:** 10.3390/ma15145080

**Published:** 2022-07-21

**Authors:** Ana Maria Lupu (Luchian), Marius Mariş, Traian Zaharescu, Virgil Emanuel Marinescu, Horia Iovu

**Affiliations:** 1 Advanced Polymer Materials Group, University Politehnica of Bucharest, 011061 Bucharest, Romania; ana.lupu@eli-np.ro (A.M.L.); horia.iovu@upb.ro (H.I.); 2 Extreme Light Infrastructure-Nuclear Physics (ELI-NP), Horia Hulubei National Institute for Physics and Nuclear Engineering (IFIN-HH), 077125 Magurele, Romania; 3 Dental Medicine Faculty, University Titu Maiorescu, 22 Dâmbovnicului Tineretului St., 040441 Bucharest, Romania; 4 INCDIE ICPE CA, Radiochemistry Center, 313 Splaiul Unirii, 030138 Bucharest, Romania; virgil.marinescu@icpe-ca.ro; 5 Academy of Romanian Scientists, 050094 Bucharest, Romania

**Keywords:** PLA, SIS, silica nanocomposite, stability, chemiluminescence

## Abstract

In this paper, the stability improvement of poly(lactic acid) (PLA)/styrene-isoprene block copolymer (SIS) loaded with silica nanoparticles is characterized. The protection efficiency in the material of thermal stability is mainly studied by means of high accurate isothermal and nonisothermal chemiluminescence procedures. The oxidation induction times obtained in the isothermal CL determinations increase from 45 min to 312 min as the polymer is free of silica or the filler loading is about 10%, respectively. The nonisothermal measurements reveal the values of onset oxidation temperatures with about 15% when the concentration of SiO_2_ particles is enhanced from none to 10%. The curing assay and Charlesby–Pinner representation as well as the modifications that occurred in the FTIR carbonyl band at 1745 cm^−1^ are appropriate proofs for the delay of oxidation in hybrid samples. The improved efficiency of silica during the accelerated degradation of PLA/SIS 30/n-SiO_2_ composites is demonstrated by means of the increased values of activation energy in correlation with the augmentation of silica loading. While the pristine material is modified by the addition of 10% silica nanoparticles, the activation energy grows from 55 kJ mol^−1^ to 74 kJ mol^−1^ for nonirradiated samples and from 47 kJ mol^−1^ to 76 kJ mol^−1^ for γ-processed material at 25 kGy. The stabilizer features are associated with silica nanoparticles due to the protection of fragments generated by the scission of hydrocarbon structure of SIS, the minor component, whose degradation fragments are early converted into hydroperoxides rather than influencing depolymerization in the PLA phase. The reduction of the transmission values concerning the growing reinforcement is evidence of the capacity of SiO_2_ to minimize the changes in polymers subjected to high energy sterilization. The silica loading of 10 wt% may be considered a proper solution for attaining an extended lifespan under the accelerated degradation caused by the intense transfer of energy, such as radiation processing on the polymer hybrid.

## 1. Introduction

The optimization of material stability is regularly achieved in several ways: preparation of hybrids [1,2], multicomponent blending [3,4,5], crosslinking [6,7], the addition of stabilizers [8,9], radiation processing [10,11] or any of their combinations. In the processing of plastics, an important role for further material stability is played by the technological parameters that influence the oxidation state and the history of products. In the cases of radiation procedures, the behavior of the substrate may be characterized by the possibility to provide reactive intermediates, free radicals that will be involved in the alternative decay pathways for several expected processes: oxidation or recombination [12]. The efficient activity of unsaturated monomers in the crosslinking of a polymer is the reason by which degrading polymers, such as PLA may gain satisfactory performances for their implementation in hazardous conditions [13]. The appropriate texture of PLA based products destined for various applications in the ranges of packaging materials or medical wear is obtained by the potential stabilizing materials belonging to the class of natural antioxidants [14,15,16]. The extension of the usage period under special environmental conditions is a general expectancy for avoiding the damage of aging materials. Accordingly, the structure of polymer backbones defines the scission positions that indicate noticeable changes occurred during degradation [17].

PLA, a biopolymer, offers multiple versions of materials destined for various application ranges: food and beverage packaging, prostheses, catheters and scaffolds, drug delivery supports, safety toys or commodities and many others. The analysis of potential applications is an attractive subject, which assists the manufacturers in the selection of compositions and technologies [18,19,20,21]. The aging behavior of PLA compounds is essentially described as depolymerization and partial oxidation [22,23]. The blending of PLA with other polymers turns the functional properties including material stability into the required features. The presence of polyhedral oligomeric silsesquioxane [24], montmorillonite and carbon nanotubes [25], and graphene [26] allows for improving the oxidative resistances of basic material. At the same time, the mixing of PLA with other polymers, for example, fulvic acid–g-poly(isoprene) [27], acrylonitrile butadiene styrene [28], polyhydroxybutyrate [29], cinnamic acid [30] represents an appropriate method for the modeling of properties. The control of product stability is the main target with which the producer is guided in the selection of raw materials [31]. The availability of PLA to generate various materials may be defined by the hundreds of combinations and associations by which the foreseen purposes are achieved. The polymer materials that are destined for ecologically friendly products must present a pertinent degradation rate so that they may be easily recycled, converted into other useful derivatives or destroyed without pollution [32]. The ability of mixed PLA-based materials to be considered biodegradable is demonstrated by their capacity to be regenerated [33], recycled [34] or transformed into ecological materials by mixing [35,36,37,38]. Therefore, our study envisages the identification of a proper system with which the manufacturers may produce appropriate items with a certain degree of stability. The manufacture of environmentally accepted materials opens several new directions for the economical implementation of PLA based blends. The mixing of PLA with polycaprolactone (PLC) offers certain possibilities for modeling materials to earn the desired properties [39,40]. The higher the content of PLA, the lower the stability of blends. The addition of triallyl isocyanate (TAIC) creates the intermolecular bridges that gather the material into a more dense and resistant compound, when the second component is poly(butylene adipate-co-terephtalate) (PBAT), a nonresistant configuration on the action of ionizing radiation [41]. The low amount of TAIC (3%) generates enough concentration of radicals for curing the two component fractions into a homogenous product.

The analysis of radiation effects on the blends consisting of PLA and EVOH [42] reveals the optimum processing dose at around 30 kGy when a great proportion of insoluble fraction (more than 80%) is formed and the mechanical properties become relevant for long term applications.

The significant energetic requirement for the modification of biopolymers limits the deterioration of polymer skeletons and extends functional qualities [43]. The electron beam (EB) irradiation creates crosslinked islands, which become efficient barriers against the diffusion of liquid and gaseous fluids. This earning is the fundamental feature for the mitigation of degradation.

**Figure 1:**
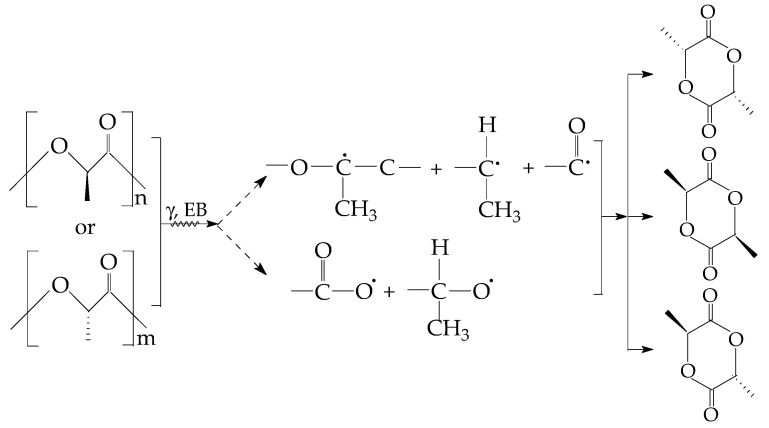
The radical intermediates that precede the formation of stable oxygenated products during degradation.

The progress of the radiation damaging of PLA is conducted by the scission tendency confirmed by the decreasing of the values of glass transition as the dose smoothly grows [44] or by the EPR assay on the modification of the abundance of radical accumulation [45]. The following degradation intermediates appear as it is illustrated in Figure 1, where the two types of radicals are formed. One kind of radical is carbon centered fragments due to the scission of C–C bonds; the second type of intermediates possess the radical positions on oxygen atoms. This last group appears from already oxidized intermediates that are the precursors of final stable products.

The evaluation of molecular modification by the gel permeation assays on EB-irradiated PLA [46] shows a sharp drop in the average molecular weight from 250 kg mol^−1^ to 30 kg mol^−1^, when the exposure dose turns from 0 to 200 kGy, while the average molecular weight drops from 260 kg mol^−1^ to 68 kg mol^−1^, under the same irradiation regime. The γ-exposure effects on PLA are based on radiochemical fragmentation, eventually followed by crosslinking. The evolution of fragmentation is described by the ratio of scission and crosslinking yields, G(S)/G(X), with values in the range of 7.6 to 10.4 [47]. Additional proof of the advanced molecular scission is the high value of 1.97 for scission yield showing that this figure characterizes the number of molecules that are broken for each 100 eV. The recent studies on the degradation of PLA [22,48] point out the essential contribution of inorganic fillers that control the level of degradation by their involvement as radical scavengers. The golden rule regarding the radiochemical yields that states to which polymer category a material belongs is also applied in the cases of PLA hybrids. In these cases, the ratio G(S)/G(X) keeps the values far from the unit (9.38 or 35.5 for two steric sorts of polymer) [49]. These figures indicate clearly that PLA has very low radiation stability and, consequently, the improvement in this feature asks for specific additives or suitable fillers that are able to convert it toward a material suitable for radiation processing or to protect it.

The PLA-based composites have gained the name of biomaterials because they are appropriate for several friendly applications from the medical area up to food packaging. The associations of this polymer with polyethylene glycol and chitosan [50], carbon [51] or pineapple [52] or pulp [53] fibers, hemp component [54], montmorillonite [55], bamboo powder [56], alginate [57] are some of the examples which confirm the versatility of PLA. These materials are potential candidates for the usage addressed to ecological products. The endurance of PLA systems is the main target for the characterization of product durability, the extension of application areas, or the creation of new materials that are able to resist damaging energy transfers [58,59]. They not only solve the problem of ecological materials but, at the same time, they also answer the requirements of healthy life.

The association between PLA and SIS in polymer formulations was formerly investigated finding that their native blends present moderate radiation stability [60]. The stability investigation of this type of blend suggests the initiation of a detailed study on the contribution of efficient additives for the integration of these materials through ecological and resistant products. The material behavior exposed to high energy radiation finds the closest applications in radiation sterilization, which is an appropriate procedure for the reduction of the bacterial charge of a large series of plastic items.

## 2. Materials and Methods

The blending components and their mixing processing for the sample preparation were presented in an earlier paper [60]. From the previous formulation of PLA/SIS compounds, the ratio 70:30 was selected for the present assay because it presents the highest radiation stability. The further nomination of material is PLA/SIS 30. This preference is the practice option for demonstrating the capacity of SiO_2_ to ameliorate the poor performances of this polymer blend. The incorporated filler, hydrophilic silica, usually incorporated in the polymer compositions, AEROSIL 200 (specific surface 200 cm^2^ g^−1^) was manufactured by Degussa (Evonic, Troy Hills, NJ, USA). For a reliable comparison of results, there were preferred four filler concentrations namely 0%, 3%, 5% and 10%. All three components, PLA, SIS and SiO_2_ were mixed together in a foreseen proportion in a Brabender Plastograph at 180 °C for 6 min with a rotor speed of 50 rpm. Then, the neat material was pressed into sheets (150 × 150 × 1 mm) under a pressure of 125 atm for 1 min after a preheating operation for 3 min at 180 °C.

The γ-exposure was accomplished at a dose rate of 0.6 kGy h^−1^ in the air inside the irradiation machinery Ob Servo Sanguis (Budapest, Hungary), which is equipped with a ^60^Co source. All the measurements were conducted immediately after the elapse of radiation treatment to avoid any unexpected modifications in the chemical states of samples. The main results of γ-irradiation were planned to explain the stability degree at the sterilization dose (25 kGy), but for the explanation of gelation and the identification of the structural differences in FTIR spectra a dose of 50 kGy was also applied.

The stability investigation is based on the chemiluminescence measurements by the nonisothermal procedure [61] at four heating rates: 3.7, 5.0, 10.0 and 15.0 °C min^−1^ and by isothermal determination [61] at 140 °C. The samples were prepared by placing polymer small sheet fragments in aluminum pans as a very thin layer for avoiding the self-absorption of CL photons. The amount of dry polymer was around 3–5 mg. The activation energies required for the thermal degradation of samples were calculated using onset oxidation temperature (OOT) values, a kinetic parameter that describes the temperature which indicates the start of oxidation. It is found at the intersection between the tangent drawn on the ascendant curve of CL intensity vs. temperature and the Ox axis. The relationship [62] (1) was applied

(1) $$\ln\left(\frac{\beta }{T^{2}}\right)=k-\frac{E}{\mathrm{RT}}$$

and allows the linear representation of ln(β/T^2^) vs. T^−1^. The slope will be used for the calculation of activation energy by dividing it by the universal gas constant, R.

The evaluation of gel content was accomplished by the measurement of the dissolved amount by boiling in Soxhlet equipment using *o*-xylene as solvent followed by drying and removing of the remaining solvent by cleaning with ethanol and the final drying. The dryings were accomplished in an electrical oven for one hour at 100 °C. The sample weights at the beginning of boiling (m_o_) and the final weight (m_f_) were obtained by analytical balance, whose error is ± 0.2 mg. The copper bags have final weights symbolized by m_b_. The soluble fraction (named sol) is calculated by Equation (2):

(2) $$S=1-\frac{m_{f}-{\;m}_{\mathrm{fo}}}{m_{b}-{\;m}_{\mathrm{fo}}}$$

The triplicates were subjected to the solubilizing step.

These values were the input values for the Charlesby–Pinner relationship [63], Equation (3), by which the corresponding graph is drawn.

(3) $$S+\sqrt{S}=\frac{G\left(S\right)}{G\left(X\right)}+\frac{1}{{\mathrm{qU}}_{o}}\;\frac{1}{D}$$

where S is sol content, D is processing dose (kGy), G(S) and G(X) are the radiochemical yields (events/100 eV) for scission and crosslinking, respectively, q denotes the crosslinking density (cm^−3^) and U_0_ is the initial polymerization degree.

The aging effect induced by γ-exposure is analyzed by the modifications that occurred in the FTIR spectra at 1745 cm^−1^ in a JASCO 4200A (Tokyo, Japan) spectrometer with a resolution of 4 cm^−1^ and 48 scans.

The SEM images were obtained with a scanning electron microscope model field emission SEM–Auriga (Carl Zeiss, Overcoached, Germany). The selected experimental parameters were: 2 kV acceleration voltages with a working distance of ~3.5 mm in a high vacuum room. Polymer samples, having been embedded in epoxy resin with conductive properties, were further polished and treated with permanganic (chemical solution) etching. The prepared surfaces were analyzed in a SESI (secondary electron secondary ions) detector chamber of the Everhart–Thornley type model.

## 3. Results

The qualification of polymer materials for their long life application asks for the identification of optimal conditions for the improvement of oxidation strength. PLA, the major component of the studied compositions, presents a very high radiochemical yield of scission (14.5) [24,64] and a very low radiochemical yield of crosslinking (0.4) [24,64] when it is irradiated in air. It undergoes thermal and radiation oxidative degradations with the activation energy placed in the range of 92–118 kJ mol^−1^ [27,55]. On the other hand, the fragmentation of D,L-PLA backbones presents a high radical yield [G(R) = 2.4 at 77 K and 1.2 at 300 K]. The radiolysis radicals (Figure 1) intimately interact with the radicals formed from SIS [60]. The presence of an oxidation inhibitor, powder POSS [65], extends the thermal stability that becomes a good material with a convenient lifespan. The certification of this processing availability is the extended ranges of usage that are based on the material accessibility for enlarging the manufacturing of the various product numbers [66,67,68]. Due to the specificity of the delaying of oxidation, the SiO_2_ fillers are capable to catch free radicals on their nanoparticle surface and, consequently, withdraw intermediates from the degradation chain [69].

### 3.1. Chemiluminescence

**Figure 2:**
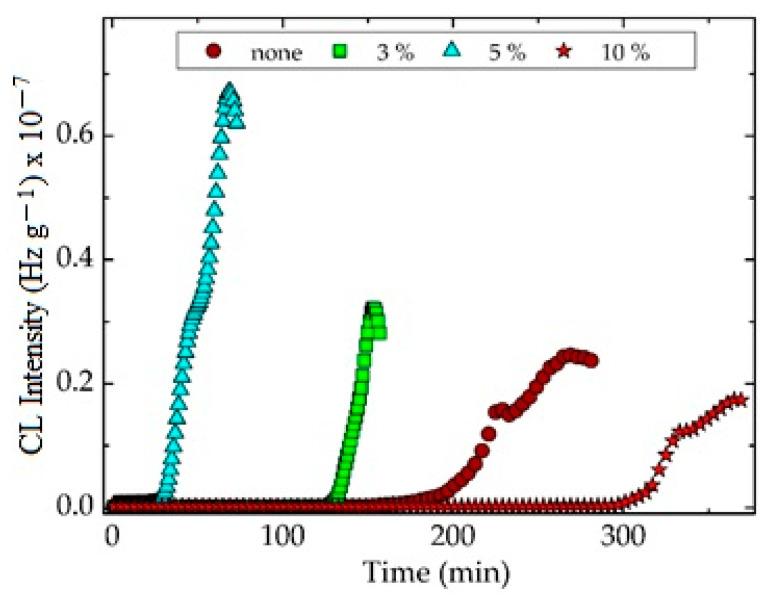
The isothermal CL spectra recorded on nonirradiated PLA/SIS 30 blends modified with silica. Testing temperature: 140 °C.

The chemiluminescence determination of the oxidation susceptibility by isothermal procedures (Figure 2) gives the measure of the stability of the attack of oxygen on the free radicals that appeared by molecular fragmentation during thermal and/or radiation degradation. The nanoparticles of silica are homogenous spread in polymer and interaction between radicals and the SiO_2_ surface is defined by the dipole–dipole attraction [70]. The increase in the silica concentration broadens the oxidation induction time. This main kinetic parameter that indicates the period over which the degradation starts presents increasing values as the silica percentage grows (Figure 2). The other feature that depicts the progress of oxidative aging is the oxidation rate. This characteristic is clearly noticed from the inclination of curves. These slopes become lower as the content of silica is higher. It means that the SiO_2_ nanoparticles act as efficient scavengers of the intermediates and break the degradation chain.

The CL spectra discover the contributions of the two mixed components which participate in the degradation with different oxidation rates. As it is known from previous studies, the degradation mechanisms of SIS [71] and PLA [72] are completely unlike. It is easy to assume that the fragments provided by the scission of SIS will catalyze the conversion of PLA into lactide. The shoulders that appeared in the CL isotheral spectra are the proof that sustains this assumption. The accumulation of CL photo-emitters takes simultaneous place from both components, but the temporal contributions follow their stability relative order. The separation of these contributions is more clearly noticed at lower temperatures when the degradation rates are larger.

The isothermal CL spectra obtained from the oxidation of unirradiated PLA/SIS 30 emphasize the two degradation sectors that correspond to the two blending components. Even though the hydrocarbon phase is degraded through the former step, when peroxyl radicals appear diluted in the PLA matrix, their concentration does not sustain the progress of oxidation inside this phase. After the longer duration of oxidation, the RO_2_**^.^** intermediates from the SIS molecules are decayed and their contribution to the degradation of the material is accordingly diminished. The reinforcing PLA with SiO_2_ nanoparticles is a pertinent solution for preventing the oxidation of radicals formed by the peroxyl phase. The implication of PLA in the accumulation of degradation products is minimized due to the lack of reactions between PLA fragments and diffused oxygen [60]. This behavior allows concluding that the permeation of oxygen drops when the amount of silica increases.

The superior thermal performances of PLA/SIS 30 filled with SiO_2_ are achieved by the existence of OH groups on the surface of particles [73]. Thus, the applications in food packaging must take into account the oxidation performances that are evidently influenced by the blending state and service conditions. The isothermal CL spectra demonstrate the earning noteworthy functionality in unfriendly environments.

**Figure 3:**
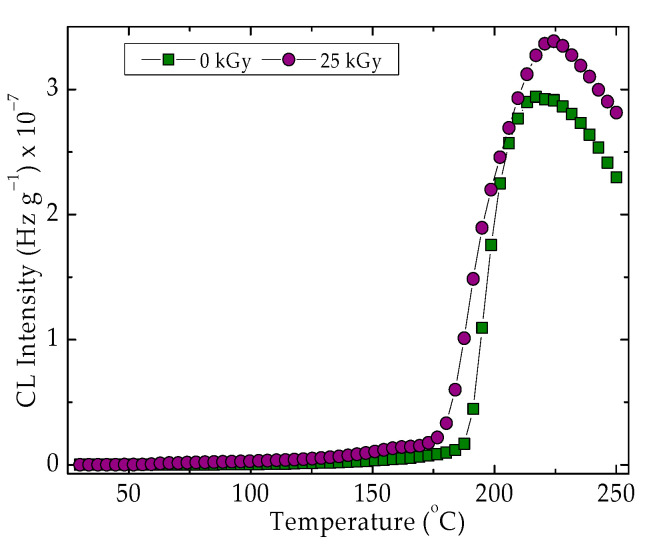
Nonisothermal CL spectra recorded on control PLA/SIS samples under different irradiation state. Heating rate: 3.7 °C min^−1^.

The exposure of the studied material to the action of γ-radiation induces a diminution in the thermal stability, especially in the range of higher temperatures [74] (Figure 3). The oxidation starts earlier and the temperature increases over 200 °C, which may induce the slight recombination of hydrocarbon radicals and cyclization of lactide structures. According to the ESR investigations on γ-irradiated PLA, the crosslinking probability is certainly higher at the temperature exceeding 100 °C due to the appropriate concentration of radicals and the greater crosslinking radiochemical yield (about 5 for the irradiation accomplished at 170 °C) concerning the lower value of scission radiochemical yield (about 1.5 under similar conditions).

The difference that exists between the radiation stabilities at the two different exposure doses (Figure 3) proves the predominant oxidation of SIS components rather than the degradation of PLA. If the PLA fragmentation is dominant, the curve shape would present a less tall peak at 225 °C.

**Figure 4:**
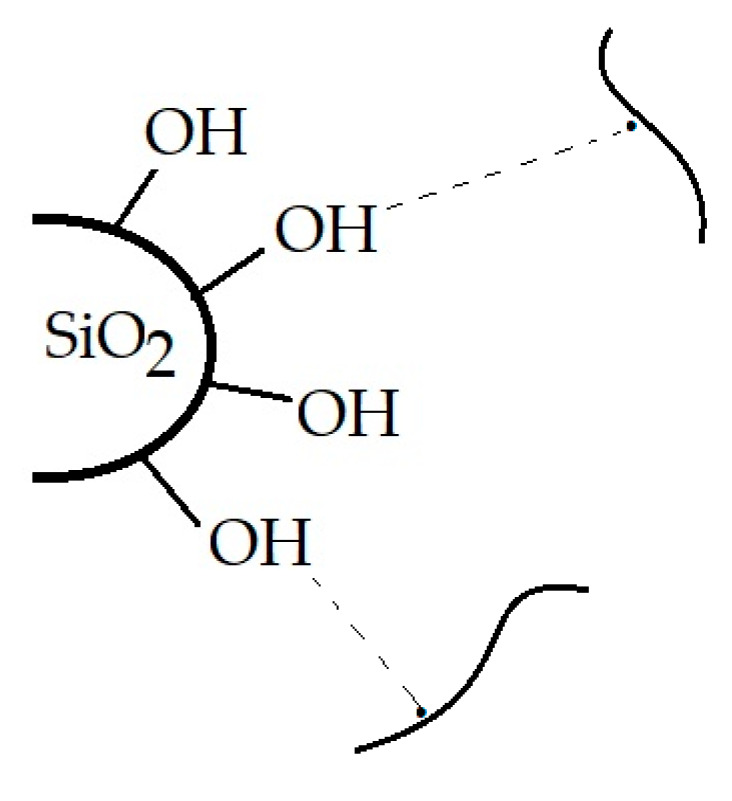
The illustration of the basic interaction between silica particles and free radicals.

Figure 4 shows the evolution of oxidative degradation on pristine materials where silica is the stabilization agent. This filled particle surface is in direct contact with the polymer substrate, creating the proper conditions for the formation of hydrogen bridges between the superficial hydroxyls and molecular fragments (Figure 4).

**Figure 5:**
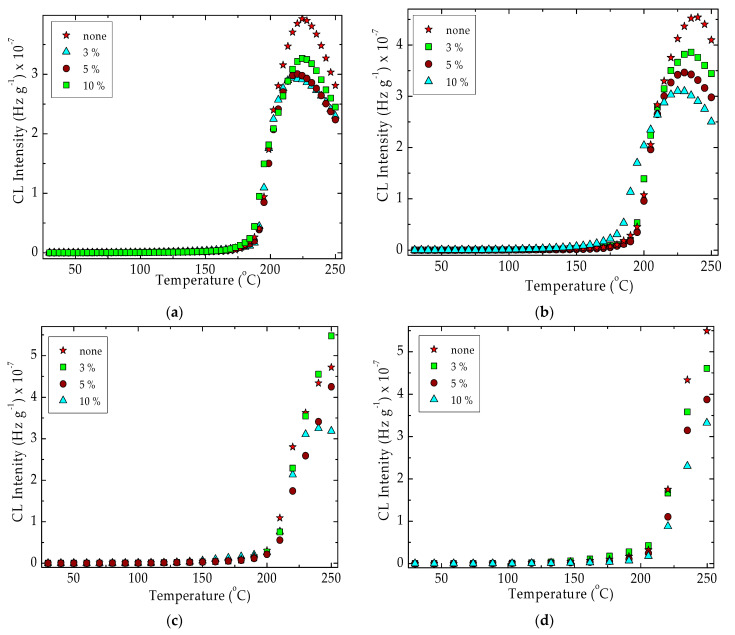
Nonisothermal CL spectra drawn for unirradiated PLA/SIS 30 in the presence of silica. Heating rates: (**a**) 3.7 °C min^−1^; (**b**) 5 °C min^−1^; (**c**) 10 °C min^−1^; (**d**) 15 °C min^−1^.

The evolution of oxidation when the temperature is rising is detailed in Figure 5. For all the heating rates, the stability sequence places the silica concentration in the following order:

none < 3% < 5% < 10%

The heating rate influences the level of oxidation mostly on the higher values of temperatures. In the case of slower heating, the oxidation peak presumably ascribed to the peroxyl radicals that appeared from the oxidation of hydrocarbon intermediates (the fragments from the scission of SIS) may be noticed at 225 °C. The higher heating rates create suitable conditions for the fast oxidation of radicals depleting them before the temperature reaches 200 °C. A specific feature that may be observed is the similitude in the oxidation degrees before 150 °C when all the samples keep constantly low CL emission intensities. The shoulder describing the formation of a significant amount of hydroperoxides as the result of the oxidation of radicals does not appear. This evolution is achieved when the degradation of polyolefins occurs in the presence of silica nanoparticles [75]. This peculiarity may be explained by the high content of PLA (70%), which does not form peroxide structures. Table 1 presents the basic values of OOT (onset oxidation temperature) when the oxidative degradation theoretically starts.

**Figure 6:**
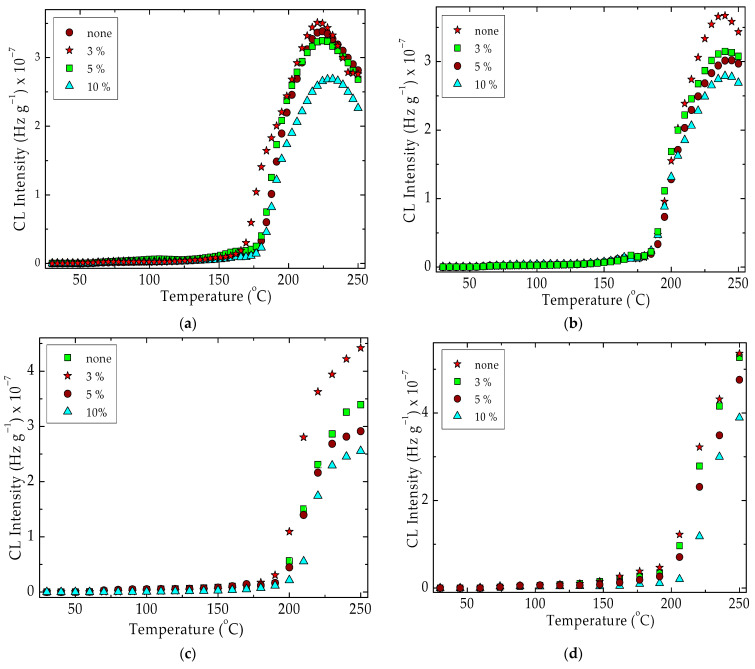
Nonisothermal CL spectra drawn for unirradiated PLA/SIS 30 in the presence of silica after receiving 25 kGy. Heating rates: (**a**) 3.7 °C min^−1^; (**b**) 5 °C min^−1^; (**c**) 10 °C min^−1^; (**d**) 15 °C min^−1^.

The γ-exposure of samples maintains similar aspects for each heating rate (Figure 5 and Figure 6). The stability order follows the same sequence, where the control is the most vulnerable material. Though this energetic treatment does not deeply modify the evolution of emission CL intensities, the OOT values are lower than for pristine samples (Table 1). The modification of the values ascribed to the oxidation onset temperatures is proof of the contribution of oxygen diffusion into the oxidizing material. The consequence of a faster increase in temperature is the real delay of oxidation due to the differences that exist between the rate of heating and the rate of diffusion. At the applied highest heating rate (15 °C min^−1^) the degradation requires higher temperatures with the unlike values for each of the silica loadings. It is possible to assume that the oxidation takes place with different diffusion rates because the components provide specific amounts of free radicals in correlation with this oxygen diffusion process.

The progress of degradation has a similar appearance for all concentrations of silica filler, but the CL curve families present unlike aspects when different heating rates are applied. It means that the presence of the descending part in the higher temperature range is proof of the competition between oxidation and the simultaneous reactions in which the intermediates are usually involved in radiolysis (disproportionation or bimolecular processes between two peroxyl radicals) [76].

The adjustable degradation is obtained if the heating rate is diminished (Figure 6, Table 1). The increase in the thermal resistances of the inspected compositions is influenced by the heating rate. This feature may be correlated with the oxygen penetration rate, which occurs arduously in the samples with higher silica loadings. As it may be observed, the higher the heating rate, the greater the onset oxidation temperatures. Simultaneously, the intensity peak recorded in the high temperature range is shifted onto the higher values and for the heating rate of 15 °C min^−1,^ it disappears. The amounts of silica existing in the sample compositions decrease the amplitude of degradation, which is indicated by the relative positions of curves one under another. The main evidence for the contribution of silica to the improvement of thermal resistance is the upper positions of CL curves recorded on the pristine compositions, where the absence of silica permits the faster progress of oxidation.

**Figure 7:**
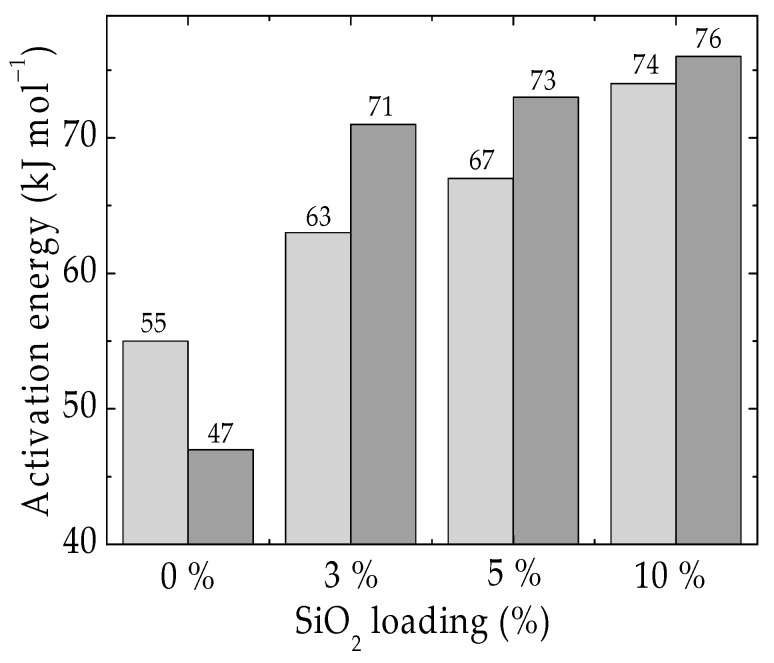
Histogram of the activation energies calculated for the oxidation of PLA/SIS/30 loaded with silica as nonirradiated samples (light grey) or the materials receiving 25 kGy (dark grey).

The results presented in Table 1, as well as in Figure 7 indicate the opportunity of sterilization processing of these materials by radiation exposure when the alteration of stability does not match. On the contrary, this treatment ameliorates the oxidation resistance to a certain extent despite the degradation effects of high energy radiation. This behavior may be associated with an earlier crosslinking due to the inhibition effect of silica on the oxidation way of molecular fragments. Therefore, this sterilization operation is suitable not only for bacterial cleaning, but also for the extension of durability.

The damage consequences in the structure of the studied materials accompanying the weakening thermal resistance are evident when these materials are exposed to sunlight or an excess of stress. The appraisal of stability has to explain the internal interaction of components.

The γ-exposure of samples maintains similar aspects for each heating rate and the stability order follows the same sequence, where the control is the most vulnerable material. Though this energetic treatment does not deeply modify the evolution of emission CL intensities, the OOT values are lower than for pristine samples (Table 1). This behavior indicated the worsening operation performances when these compounds are subjected to an intense degradation process, such as γ-treatment or UV-exposure.

The OOT values are the input data for the calculation of the activation energies required by polymer samples by the Kissinger procedure [62]. As it is foreseen for the radiation affected hybrid, the activation energy is lower for the unmodified polymer sample, but the corresponding values for hybrids are significantly higher (Figure 7). The protection activity of silica is related to the catching of intermediates by the oxygen atoms existing on the nanoparticle surface. The demonstration of this behavior was presented by the grafting of (3-aminopropyl)triethyxysilane on the silica surface [77,78]. The higher energetic requirements suggest the effectiveness of the filler contribution to the delay of oxidation that recommends this neutral additive for the extension of material durability in risky applications.

The stabilization effect induced by the presence of silica on the composition of PLA hybrids was also reported [79]. The present values are lower than 100 kJ mol^−1^ found for the degradation of PLA by the conversion procedure because our materials contain a radiation degradable structure, namely SIS. However, these satisfactory values displayed are reliable proofs for foreseen purposes, such as materials for food packaging or safety preservation during shipment. The already aforementioned comments highlight the strengthening effect that leads to the improved operation life of these materials and the lack of danger.

### 3.2. Gelation

**Figure 8:**
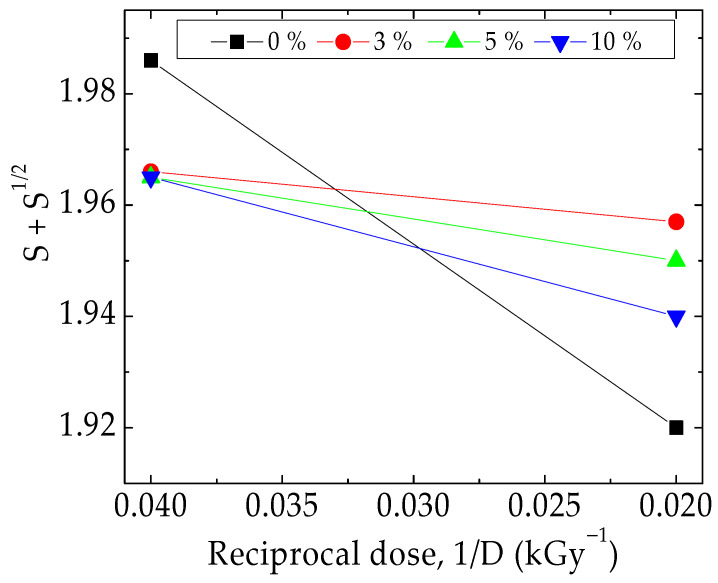
Charlesby–Pinner representation of radiation effects in PLA/SIS 30/SiO_2_ hybrids.

By the energy transfer on the macromolecules of the PLA/SIS 30 compound, the radiation processing modified with silica is the determinant cause of the fragmentation of the macromolecules, generating free radicals. According to the radiolysis mechanisms, they may be decayed by oxidation or recombination. The decrease of the soluble fraction (sol, S) is the measure of the inter-radical reactions and indicates the gelation of the material. In Figure 8 there is a visible difference between the material without silica and the samples containing this inorganic compound. While the pristine blend is sharply damaged, the hybrids present slight gelation due to the contribution of hydrocarbon radicals formed from the SIS structure. The contribution of silica to the protection against oxidation is defined by the slope of lines. The greater the slope, the larger amount of insoluble fraction is formed.

### 3.3. FTIR Evaluation

**Figure 9:**
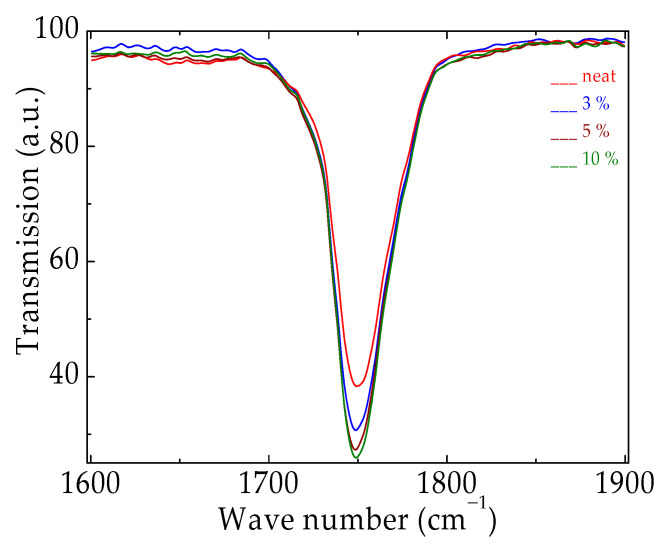
The FTIR spectra recorded on the PLA/SIS 30 modified with nanoparticles of silica after their exposure to a γ dose of 50 kGy.

The spectral assay on the oxidation state after the γ-exposure of PLA/SIS 30 in the presence of silica is presented in Figure 9. Even though the PLA molecules contain oxygen and their scission would generate oxygen-centered radicals, this contribution represents a spectral background that is similar to all the inspected compositions. The differences that exist are found in the differences in transmission values due to the oxidation process sustained by hydrocarbon radicals. As may be noticed from Figure 9 the accumulation level of carbonyl products decreases with the concentration of silica. As the CL determinations already showed, the silica loading brings about certain protection against oxidation, which is delayed proportionally with filler assisting components.

If it is taken into consideration that all studied compositions have similar FTIR spectra, the image offered of the progress of oxidation is accurately appraised by the differences in the transmission figures.

### 3.4. SEM Assay

**Figure 10:**
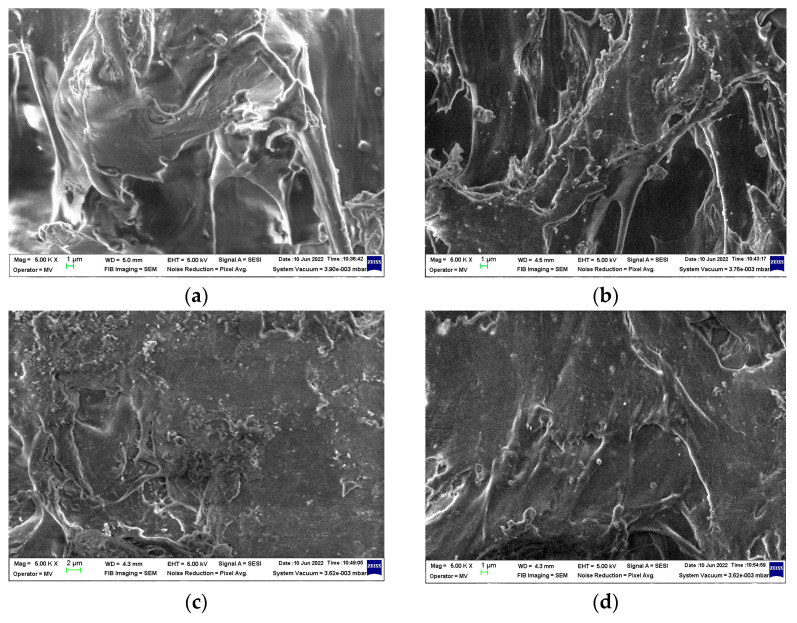
The SEM images on nonirradiated PLA/SIS 30 samples. Compositions: (**a**) control; (**b**) SiO_2_ 3%, (**c**) SiO_2_ 5%, (**d**) SiO_2_ 10%. Magnification: 5 × 10^3^.

The SEM investigation of the PLS/SIS 30 samples reveals the homogenization of the polymer substrate (Figure 10). The clear separation of silica particles may be noticed when the surrounding polymer becomes the proper material for product modeling. The filler is homogenously dispersed and the morphology shown by these micrographs demonstrates the availability to form composites as was reported earlier [80].

**Figure 11:**
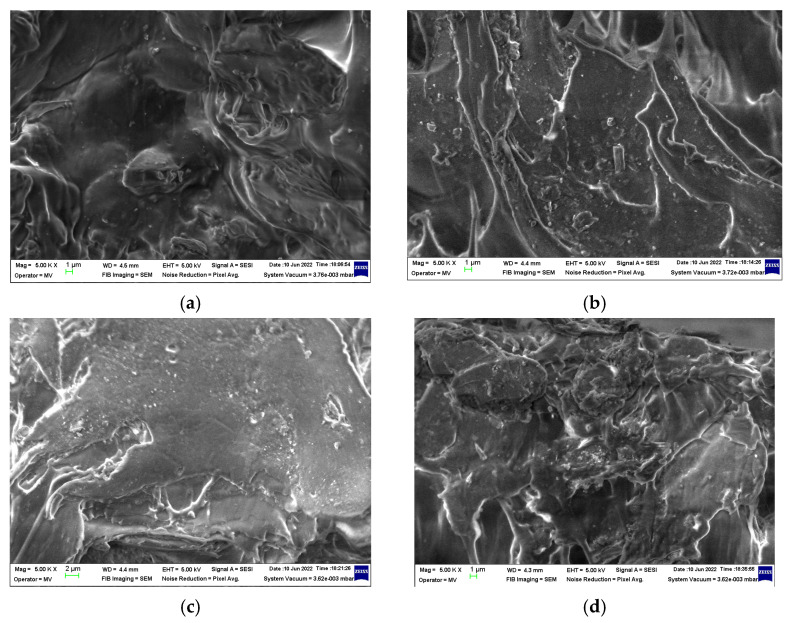
The SEM images on PLA/SIS 30 samples irradiated at 25 kGy. Compositions: (**a**) control; (**b**) SiO_2_ 3%, (**c**) SiO_2_ 5%, (**d**) SiO_2_ 10%. Magnification: 5 × 10^3^.

It would be expected that γ-irradiation, the process which initiates the damage of macromolecules, affects the microstructures of samples. However, the dominant component, PLA, subjected to γ-radiolysis follows a degradation mechanism through which its molecules drop down their macrostructures, but the resulting fragments are oxidized to lactides. As it can be noticed from Figure 11, the curing feature is demonstrated. It means that the stabilization action of silica particles is an important factor that promotes a slight crosslinking as the comparison of corresponding images illustrates. Therefore, the presence of silica in the PLA/SIS 30 samples makes the compaction in the polymer phase possible. The filler particles are clearly separated by organic materials, where scissions occur to feed the crosslinking process. The free radicals nearby particle surfaces may interact easily with hydroxyls belonging to silica (Figure 4) hindering their coalescence. The great advantage of this presence is the prevention of material cleavage which would influence the penetration of oxygen. The integrity of samples after γ-irradiation is a consequence of the perfect compatibility of mixed components.

## 4. Discussion

The silica/polymer composites show convenient properties related to the processability or spectrum of applications. The covalent interfacial connections between silica particles and polymer substrate are the reason for the appropriate performances of these hybrids [81,82]. The nanotechnologies approaches provide the specific solutions for the formulations of resistant materials, which are based on improving the contribution of inorganic fillers [83,84]. Various formulations based on PLA try to find optimal solutions for the enlargement of application areas: biodegradability [74,85,86], medical range [87,88], packaging products [89,90], optical bioimaging [91], controlling the release of hydrophilic pharmaceutical compounds [92], production of contractible materials [93], recycling [94].

The addition of silica in the polymer matrices is a proper way to improve the main characteristics of these materials [95,96,97]. This statement is based on the interaction between the polar intermediates and the hydroxyls existing on the surface of filler particles. The evolution of degradation is correlated with the contributions provided by components, especially by SIS, whose scission process initiates a radical mechanism. However, the stability aspects describing the progress of oxidative degradation are often disregarded, because the assays are axed on the functional properties and the relation between the composition and the consequences of external factors on the material behavior, for example [98,99,100]. The customers that are the beneficiaries of research need the stability characterization of materials that they purchase based on the present results concerning the activation energies involved in the stabilization of these types of substrates. Accordingly, the intimate interaction of the two phases (organic and inorganic ones) may be assessed by the increase of stability by about 20–25% due to the presence of silica (Figure 7). It means that the durability tested with the accelerated degradation by γ-exposure is significantly augmented and the product lifetimes are substantially extended.

As would be expected from other previous investigations [101], the thermal performances of PLA/SIS 30 hybrids with silica are influenced by the concentration of filler (Figure 2). The presence of silica whose structure allows intimate connections with the radical fragments by means of hydroxyl bridges brings about a visible increase to the values of oxidation induction time. This essential kinetic parameter becomes 128 min, 210 min and 320 min for the SiO_2_ concentration of 3 wt%, 5 wt% and 10 wt%, respectively, while it remains 36 min for pristine composition. Thus, the silica nanoparticles efficiently scavenge the degradation intermediates that delay the aging process by interfacial interactions.

The nonisothermal spectra (Figure 5 and Figure 6) are appropriate proof confirming the prevention effect of silica on the progress of oxidation in PLA based compounds as was presented in other previous papers [102,103]. This behavior is in contrast with the instability of polyolefins [77], which present prominent CL intensity maxima at 100 °C by the abundant formation of hydroperoxides. The thermal characteristics of the major component, PLA, in the presence of silica particles (melting point around 155 °C and Tg is placed between 53 and 63 °C [104]) are modeled and a sharp increase of CL intensity over 160 °C proves the contribution of the nanofiller phases. The differences that exist appear at higher temperatures when the thermal diffusion is more pronounced allowing a closer nearness of polymer fragments to the silica nanoparticles. These dissimilarities shown by the OOT values (Table 1) demonstrate that this filler acts by the attraction of oxidizing intermediates. The higher loading of silica shows the most efficiency in the prevention of oxidative degradation.

The analysis of the activation energy values (Figure 6) offers the possibility to estimate the interaction depth between material components, whose concentrations indicate a strong dependence. As the loading of silica increases, the values of Ea become higher. This improvement is tightly related to the formation of active intermediates, which keep the surface of silica particles and the forming radicals closed. It limits the progress of oxidation by withdrawing intermediates from the degradation chain during the propagation stage. The γ-exposure decreases the required values of activation energies because the competition between each free radical diminishes the probability of protective hindering and the availability of active radicals for their oxidation is more extended. Always, radiolysis is a source of radicals, because, in the initiation stage of degradation, the transferred energy from the incidental radiation onto the substrate exceeds the bond energies.

The augmentation in the thermal resistance of polymers by the activity of silica is correlated with the formation of hydroxyls that are formed on the nanoparticle great surface (Figure 4) as the result of the reaction of protons generated by the degradation of the polymer and the superficial oxygen atoms that are incorporated in the filler structure [102,103,104,105] or by local hydrolysis where water molecules are formed during radiolysis [106].

**Figure 12:**
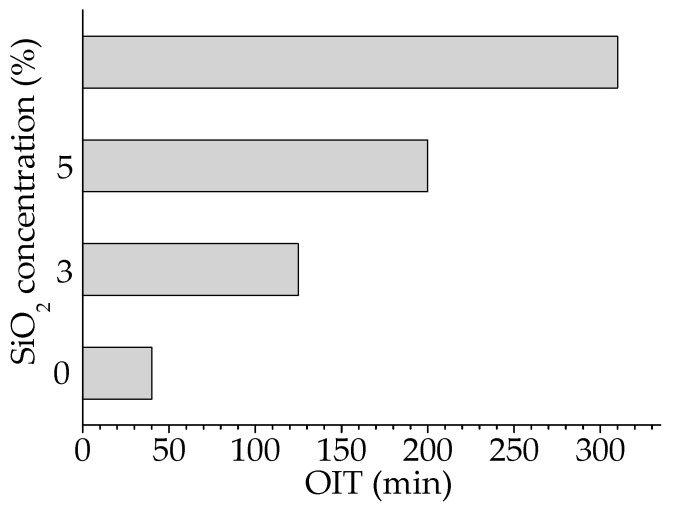
The oxidation induction times of the oxidation of PLA/SIS 30/SiO_2_ hybrids obtained by the isothermal CL measurements at 140 °C.

The progress of degradation may be accurately characterized by the measurements of CL emission that watch the accumulation of oxidation intermediates. If the isothermal version of this procedure is applied to the description of filler contribution to the initiation and propagation of oxidation (Figure 2), when the oxidation induction time (OITs) increases by ten times from 35 min to 322 min for the raw blend to the composition containing 10 wt% silica, the nonisothermal measurements allow the detailed comparison of the start-point of oxidation and the acceleration of degradation by the increase of temperature (Figure 5 and Figure 6). Thus, the thermal regimes define the manner by which the silica nanoparticles may delay the oxidation by the breaking degradation mechanism. The isothermal CL spectra demonstrate that the increase in the silica loading brings about a significant extension of induction time, the period when the material is not sensitively altered. Therefore, for the unirradiated samples, the oxidation induction time increases by 3.2 times, when the concentration of SiO_2_ is 3%, by five times as the SiO_2_ loading is 5% and by about nine times if the content of SiO_2_ is 10% (Figure 12). The evolution of oxidation is tightly related to the permeation degree [107]. This diffusion feeds the polymer bulk sustaining the progress of degradation. One procedure for the diminution of the penetration of oxygen into a polylactide is the grafting of organosilane on a silica nanoparticle surface when an efficient barrier is formed [108].

The evolution of oxidation is evidenced by the increasing transmission values for the carbonyl band. The accumulation of ketonic structures is possible if an appropriate hindrance factor does not exist. Despite the depolymerization of the polylactide component occurring during γ-irradiation [45,109], the fragments provided by the scission of the SIS component, a hydrocarbon structure, generate vulnerable radicals able to be oxidized if there is not any protection [12,110]. The nanoparticles of silica act obviously, generating free radicals and catching them on their surface. Consequently, their conversion into oxygen-containing products is obstructed and the transmission increases slowly.

This augmentation is the effect of the lack of any protection factor, even though the compound is not an authentic antioxidant; the stabilization action is based on the formation of hydroxyl moieties as a consequence of oxidation [111]. In the degradation mechanism, the competition between oxidation and protection is won by the material stability where the content of silica is high enough to withdraw radicals from their oxidation chain.

The accelerated degradation caused by the exposure of patterns to the damaging action of γ-radiation advances much slower in the processed material with increased silica loadings. The energetic conditions shown in Figure 7 demonstrate the narrowing differences in the values of activation energies. While the pristine composition has lower activation energy for its oxidative degradation after γ-treatment, the materials containing various amounts of filler present higher Ea. This behavior indicates the protection activity of silica particles with respect to the molecular fragmentation of macromolecules. This effect may be ascribed to the withdrawal of free radical intermediates from the oxidation route via the formation of hydroperoxides [112]. The stability study of the hemp-PLA composites [113] reports the activation energies for the thermal degradation of the investigated compositions in the range of 159–163 kJ mol^−1^; the PLA hybrids stabilized with POSS present activation energies between 80 and 100 kJ mol^−1^; the presence of SIS decreases this parameter due to its relative low instability [60].

The mechanistic approach must take into account the stability differences that exist between the components, which influence the fates of generated radicals. While the hydrocarbon structure of SIS is oxidized by the formation of hydroperoxides followed by their second stage reactions of conversion into oxygen-containing compounds [76], the polylactide is decomposed by depolymerization [29] and the CL emission is not recorded. The damage to this component is unlikely to be associated with the formation of lactide monomers, which sustains oxidation without the formation of ketonic structures as CL emitters. The degradation progresses does not concern the two polymers, but the contributions of intermediates of both former structures follow dissimilar ways.

**Figure 13:**
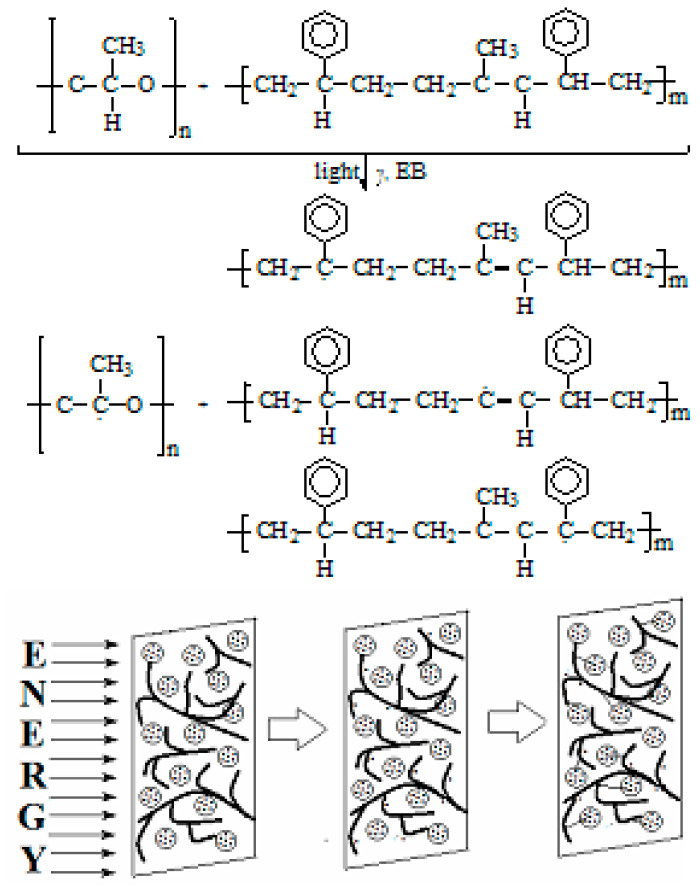
The illustration of pathway through which the radicals and silica particles interact in the PLA/SIS samples.

A conclusive illustration of the essential steps that run through the irradiated PLA/SIS filled with silica particles is offered in Figure 13.

## 5. Conclusions

This paper analyses the improvement of thermal resistance in the cases of PLA/SIS 30/n-SiO_2_ hybrids. The protection action is based on the interaction between inorganic and organic phases at the boundary zone due to the bridges established by hydrocarbon free radicals on the oxygen atoms belonging to the external layer of silica nanoparticles. The increase in silica loadings up to 10% extends the peroxidation periods which are illustrated by the growing oxidation induction time shown in the isothermal CL determinations. The measure of stabilization efficiency is suggested by the increasing values of activation energy of the oxidative degradation of polymer matrices as the amount of SiO_2_ is enhanced. While this value decreases for the pristine blend after an accelerated degradation by γ-exposure, the energetic requirements of material degradation become higher. It demonstrates that the blend free of filler is unprotected, but the presence of silica keeps the unchanged structure of the materials. The appropriate proof for the contribution of silica to the protection of polymer samples is the behavior of the irradiated specimens subjected to radiation induced crosslinking. The Charlesby–Pinner representation reveals the advantageous contribution of silica to the promotion of radical recombination instead of their reactions with molecular oxygen that diffuses into the materials during radiation processing. The variation of the carbonyl band for the irradiated samples is evidence of the scavenging efficiency of silica concerning the decaying intermediates. This insight gathering all the pertinent arguments indicates nanoparticles of silica as an appropriate formulation component of plastic products that show an improved resistance against oxidation even in the harmful conditions of accelerated degradation caused by γ-irradiation and thermal degradation.

These compositions may be properly chosen for the production of commodities, such as flexible medical wear, automotive accessories, packaging sheets, protection covers, and sealant gaskets due to their adaptableness to severe environmental conditions.

## Figures and Tables

**Table 1:** OOT values for the oxidation of PLA/SIS blends reinforced with SiO_2_ nanoparticles obtained by nonisothermal chemiluminescence.

SiO_2_ Loading (%)	OOT (°C)			
	3.7 °C min^−1^	5.0 °C min^−1^	10.0 °C min^−1^	15.0 °C min^−1^
D = 0 kGy				
0	170	175	177	187
3	175	190	205	210
5	177	191	208	215
10	187	192	210	218
D = 25 kGy				
0	150	165	184	192
3	157	168	181	186
5	162	175	185	192
10	172	181	195	202

## Data Availability

Not applicable.
